# Facile Synthesis, Characterization, Photocatalytic Activity, and Cytotoxicity of Ag-Doped MgO Nanoparticles

**DOI:** 10.3390/nano11112915

**Published:** 2021-10-30

**Authors:** ZabnAllah M. Alaizeri, Hisham A. Alhadlaq, Saad Aldawood, Mohd Javed Akhtar, Mabrook S. Amer, Maqusood Ahamed

**Affiliations:** 1 Department of Physics and Astronomy, College of Sciences, King Saud University, Riyadh 11451, Saudi Arabia; zalaizeri@ksu.edu.sa (Z.M.A.); SDawood@ksu.edu.sa (S.A.); mahamed@ksu.edu.sa (M.A.); 2 King Abdullah Institute for Nanotechnology, King Saud University, Riyadh 11451, Saudi Arabia; mjakhtar@ksu.edu.sa; 3 Department of Chemistry, College of Sciences, King Saud University, Riyadh 11451, Saudi Arabia; msamer@ksu.edu.sa

**Keywords:** MgO nanoparticles, Ag doping, structural characterization, photocatalytic activity, cytotoxicity

## Abstract

Due to unique physicochemical properties, magnesium oxide nanoparticles (MgO NPs) have shown great potential for various applications, including biomedical and environmental remediation. Moreover, the physiochemical properties of MgO NPs can be tailored by metal ion doping that can be utilized in photocatalytic performance and in the biomedical field. There is limited study on the photocatalytic activity and biocompatibility of silver (Ag)-doped MgO NPs. This study was planned for facile synthesis, characterization, and photocatalytic activity of pure and silver (Ag)-doped MgO NPs. In addition, cytotoxicity of pure and Ag-doped MgO NPs was assessed in human normal umbilical vein endothelial cells (HUVECs). Pure MgO NPs and Ag-doped (1, 2, 5, and 7.5 mol%) MgO NPs were prepared via a simple sol-gel procedure. X-ray diffraction (XRD), scanning electron microscopy (SEM), transmission electron microscopy (TEM), Fourier transform infrared (FTIR), photoluminescence (PL), and X-ray photoelectron spectroscopy (XPS) were used to characterize the prepared samples. XRD results showed the preparation of highly crystalline NPs with no impurity peaks. TEM and SEM studies indicate smooth surfaces with almost spherical morphology of MgO NPs, and Ag-doping did not change the morphology. Elemental composition study suggested that Ag is uniformly distributed in MgO particles. Intensity of the PL spectra of MgO NPs decreased with increasing the concentration of Ag dopants. In comparison to pure MgO NPs, Ag-MgO NPs showed higher degradation of methylene blue (MB) dye under UV irradiation. The improved photocatalytic activity of Ag-MgO NPs was related to the effect of dopant concentration on reducing the recombination between electrons and holes. Cytotoxicity studies showed good biocompatibility of pure and Ag-doped MgO NPs with human normal umbilical vein endothelial cells (HUVECs). These results highlighted the potential of Ag-doped MgO NPs in environmental remediation.

## 1. Introduction

Metal oxide nanoparticles (NPs) have been used for various applications including electronics, catalysis, and biomedicines [1,2]. Studies are now focusing on the use of appropriate transition metal ions to improve the photocatalytic activity of metal oxide NPs [3]. Recently, magnesium oxide (MgO) has received great attention in a wide range of applications including catalytic [4], biomedical [5], and electronic [6,7] due to its unique physicochemical properties, inexpensive synthesis, and low toxicity [8].

Physiochemical properties of MgO NPs can be further tailored by metal ion doping, which can be utilized in photocatalysis and biomedical applications. Different methods have been reported for the synthesis of metal ion doped MgO NPs. For example, synthesis of Ag-doped MgO NPs via successive ionic layer adsorption and reaction (SILAR) [9], Ce-doped MgO NPs by sol-gel [10], and Cu-doped MgO NPs by self-propagating combustion [11]. Parvizi and coworkers observed that Zn doping decreased the band gap energy and increased the optical activity of MgO [12]. Earlier studies showed that the photocatalytic properties of MgO NPs are lesser than TiO_2_ NPs. However, transition metal ion doping can improve these characteristics of MgO NPs for several applications [10,13,14]. Ag-doping also enhanced the antibacterial activity of MgO NPs [15].

Studies on the photocatalytic and biological activity of MgO NPs are gaining momentum [16]. The photocatalytic activity of MgO NPs and metal ion doped MgO was evaluated by degradation of MB dye under UV irradiation [17,18]. MgO NPs also act as bioactive compounds with several applications such as antibacterial and antioxidant [19,20]. There is limited knowledge on the photocatalytic activity and biocompatibility of Ag-doped MgO NPs. Keeping this view, the present study was designed to investigate the effect of Ag doping on physicochemical properties, photocatalytic activity, and biocompatibility of MgO NPs. Pure and Ag-doped (1%, 2%, 5%, and 7.5 mol %) MgO NPs were synthesized by a facile sol-gel method. Prepared samples were characterized by X-ray diffraction (XRD), scanning electron microscopy (SEM), transmission electron microscopy (TEM), Fourier transform infrared spectroscopy (FTIR), photoluminescence (PL), and X-ray photoelectron spectroscopy (XPS). Photocatalytic activity of pure and Ag-MgO NPs was examined by the degradation of methylene blue (MB) dye under UV irradiation. Additionally, the cytotoxicity of prepared samples was examined in human normal umbilical vein endothelial (HUVE) cells.

## 2. Materials and Methods

### 2.1. Materials 

Magnesium nitrate hexahydrate Mg (NO_3_)_2_·6H_2_O, silver nitrate (AgNO_3_), and citric acid monohydrate (C_6_H_8_O_7_·H_2_O) were purchased from Sigma-Aldrich (St. Louis, MO, USA) and used as precursors for the synthesis of pure and Ag-MgO NPs. The preparation medium was double distilled water (DDW).

### 2.2. Preparation of Pure and Ag-Doped MgO NPs

Pure and Ag-doped MgO NPs were prepared by a simple sol-gel method. The 9.98 mmol of Mg (NO_3_)_2_·6H_2_O and a varying amount of AgNO_3_ (1, 2, 5, and 7.5 mol%) were dissolved in 20 mL of double distilled water. This solution was magnetically stirred for 20 min to get a homogenous solution. Then, a solution containing citric acid (C_6_H_8_O_7_·H_2_O, 9 mmol) was added dropwise into the above reaction mixture under continuous stirring to avoid agglomeration of the particles. The mixture was placed in a water bath at 75–80 °C for 4 h to obtain a gel. This gel was washed by distilled water three times to eliminate the impurities and dried in an oven at 100 °C for 24 h to get a white or yellow precursor. Finally, the precursor was crushed and calcined at 600 °C for 3 h to yield powder samples.

### 2.3. Characterization of Pure and Ag-Doped MgO NPs

The prepared samples were characterized using XRD, FETEM, FESEM, FTIR, XPS, and PL techniques. Phase-purity and crystallinity were examined by X-ray diffraction (XRD) (PanAnalytic X’Pert Pro, Malvern Instruments, Malvern, UK) with Cu-Kα radiation (λ = 0.15405 nm, at 45 kV and 40 mA) and an angle range from 30° to 80°. Morphological and elemental mapping were identified using field emission transmission electron microscopy (FETEM, JEM-2100F, JEOL, Inc., Tokyo, Japan) and field emission scanning electron microscopy (FESEM, JSM-7600F, JEOL, Inc. Tokyo, Japan). Energy-dispersive X-ray spectrometry (EDS) was employed to determine the elemental composition. The chemical states of elements were investigated by X-ray photoelectron spectroscopy (XPS) (PHI-5300 ESCA PerkinElmer, Boston, MA, USA). Photoluminescence (PL) spectra were measured by a fluorescent spectrometer (Hitachi F-4600). Fourier transform infrared spectroscopy (FTIR) (PerkinElmer Paragon 500, Woonsocket, RI, USA) was used to identify the functional group of the prepared samples.

### 2.4. Photocatalytic Activity Measurement 

The photocatalytic activity of pure and Ag-doped MgO NPs was tested by measuring the degradation of methylene blue (MB) dye under UV irradiation. The 10 mg of MgO and Ag (1%, 2%, 5%, and 7.5 mol%)-doped MgO NPs were added to a stock solution of methylene blue (MB) (1 mg/100 mL) under constant stirring for half an hour in the dark at room temperature to reach adsorption and desorption equilibrium. The prepared solution was irradiated using a UV light source. At regular intervals of 30 min, the 2 mL solution was taken during the process of irradiation and centrifuged at 10,000 rpm for 5 min to remove nanoparticles from the solution [20]. The degradation of MB dye was measured from the absorbance at 300–800 nm by a Hitachi U-2600 UV–visible spectrophotometer. The degradation efficiency of MB can be obtained from the following equation by [21,22]:

(1) $$\mathrm{Degradation}\;\mathrm{efficiency}\;\left(D\%\right)=\;\frac{A_{0\;}-A_{t\;}}{A_{0\;}}\;\times 100\%$$

where *A*_0_ is the initial absorbance before irradiation, and *A*_t_ is the absorbance after irradiation for each time.

### 2.5. Cell Culture and Cytotoxicity Assay

The human umbilical vein endothelial cells (HUVECs) were purchased from ATCC (ATCC, Manassas, VA, USA), USA. The HUVEs were cultured in Dulbecco Modified Broker Eagle medium (DMEM) with 10% fetal bovine serum and 100 U/mL penicillin–streptomycin. Cells were maintained in an incubator with a 5% CO_2_ supply at 37 °C. The HUVECs cultured were employed in an in vitro study of medical applications. A stock suspension (1 mg/mL) of pure and Ag-MgO NPs was prepared in a culture medium (DMEM + 10%FBS). Different dilutions (5, 10, 25, 50, 100, and 200 µg/mL) were sonicated in a water bath for 20 min to prevent agglomeration of NPs before exposure to cells. Cytotoxicity of pure MgO and Ag-MgO NPs was examined by the MTT assay [23] with some modifications [24].

## 3. Results and Discussion

### 3.1. X-ray Diffraction (XRD) Analysis

**Figure 1:**
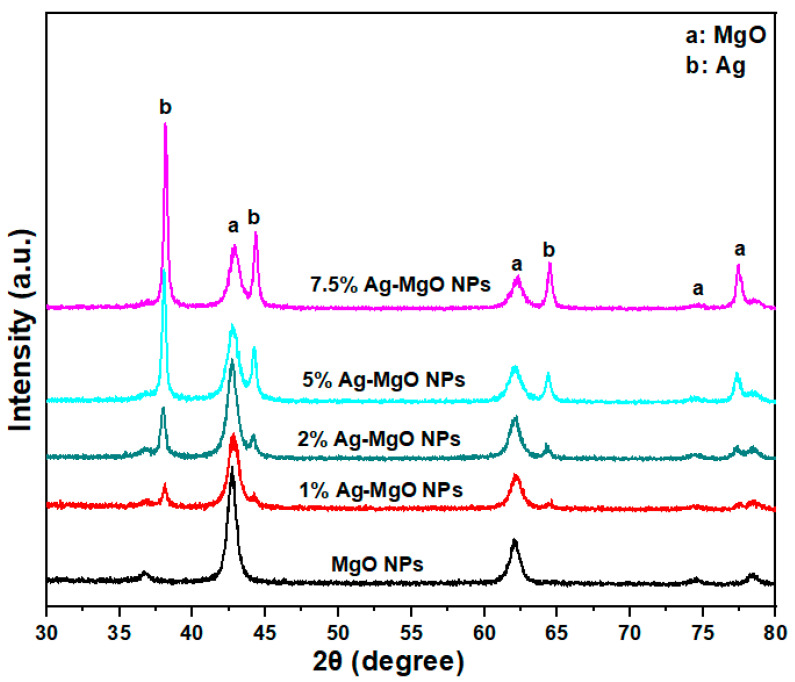
XRD spectra of pure and Ag-doped (1–7.5%) MgO NPs.

X-ray diffraction was carried out to investigate the phase purity, crystal structure, and particle size of prepared samples. Figure 1 shows the XRD spectra of pure MgO NPs and Ag (1%, 2%, 5%, 7.5 mol%)-doped MgO NPs. The position of the first peak of pure MgO and Ag (1%, 2%, 5%,7.5 mol%)-doped MgO NPs is at 2θ values 42.69°, 42.75°, 42.78°, 42.81°, and 42.91°, respectively. This slight shifting of peaks to a higher angle could be due to integration of Ag in MgO NPs crystals. This phenomenon was also reported by other investigators [9,15,25]. Presence of Ag peaks in Ag-doped MgO NPs is due to the fact that the ionic radii of Ag^+^ (0.126 nm) is larger than the ionic radii of Mg (0.066 nm) [26,27]. Further, the crystallite size (*D*) of prepared samples was calculated by Scherrer’s equation, which is given by Equation (2) [28].

(2) $$D=\frac{k\lambda }{\beta \;cos\theta }$$

where *k* = 0.90 is the shape factor, *λ* is the wavelength, *β* is the full width at half-maximum (FWHM), and θ is the reflection angle. The crystallite size of pure MgO NPs, Ag (1%, 2%, 5%, and 7.5 mol%)-doped MgO NPs was 12 nm, 11.5 nm, 11 nm, 9.5 nm, and 9 nm, respectively (Table 1). The crystallite size of MgO NPs decreased with the increasing concentration of Ag dopant. Reduction in crystallite size could be due to the crystallite size influence or surface stress in the crystal structure with the dopant Ag [29]. Such a phenomenon was also reported by other studies [30,31]. The crystal size and shape of NPs are important factors for photocatalytic activity and cytotoxicity studies.

### 3.2. Transmission Electron Microscopy (TEM) Analysis

**Figure 2:**
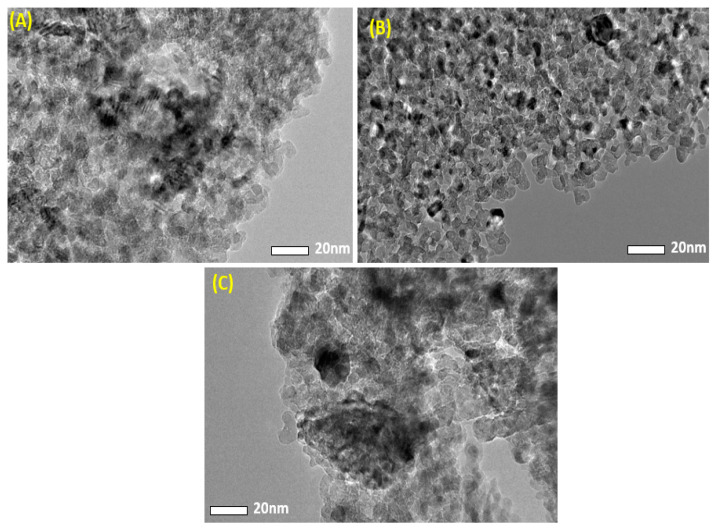
TEM images of pure MgO (**A**), 1% Ag-MgO NPs (**B**), and 7.5%Ag-MgO NPs (**C**).

The morphological characteristics of obtained samples were assessed by FETEM. Figure 2 depicts the typical TEM images of pure MgO NPs and Ag-MgO NPs. Table 1 shows that the addition of Ag in MgO reduces the average particle size. This decrease in the average particle size is mostly due to Ag dopant inhibition on crystal growth of MgO. Similar results were also reported by other investigators [32,33].

### 3.3. Energy-Dispersive X-ray (EDX) Analysis

**Figure 3:**
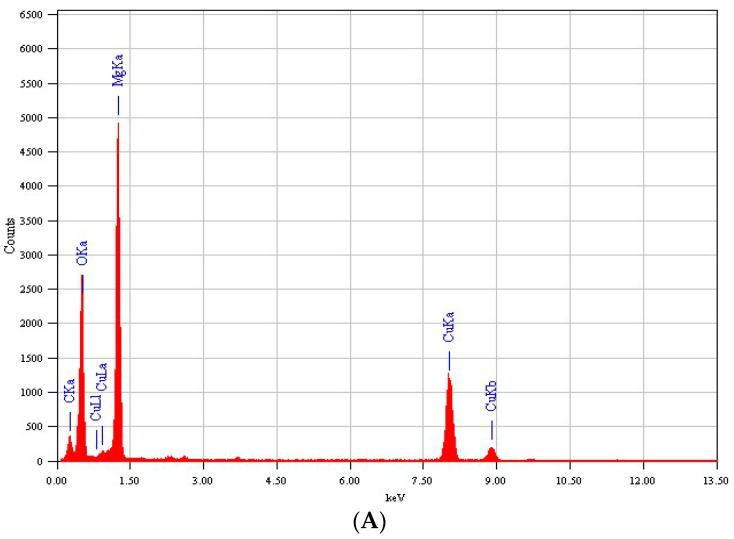

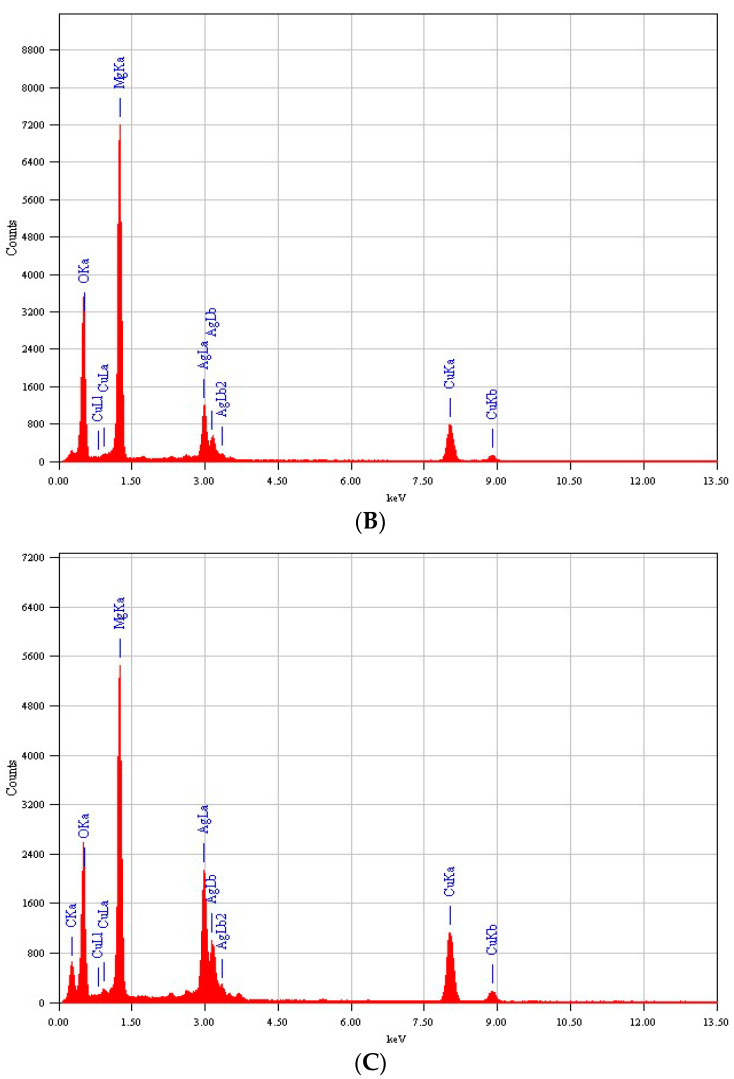
EDX spectra of pure (**A**), 5% Ag-MgO NPs (**B**), and 7.5% Ag-MgO NPs (**C**).

Energy-dispersive X-ray (EDX) spectroscopy was employed to confirm the chemical composition of the prepared sample. Figure 3 shows the clear peaks of Mg, Ag, and O. Presence of carbon (C) and copper (Cu) peaks was due to use of a carbon-coated copper TEM grid [34]. It was also noted that the peak intensity of Ag increases with increasing concentration of dopant Ag [35].

### 3.4. Scanning Electron Microscopy (SEM) Analysis

**Figure 4:**
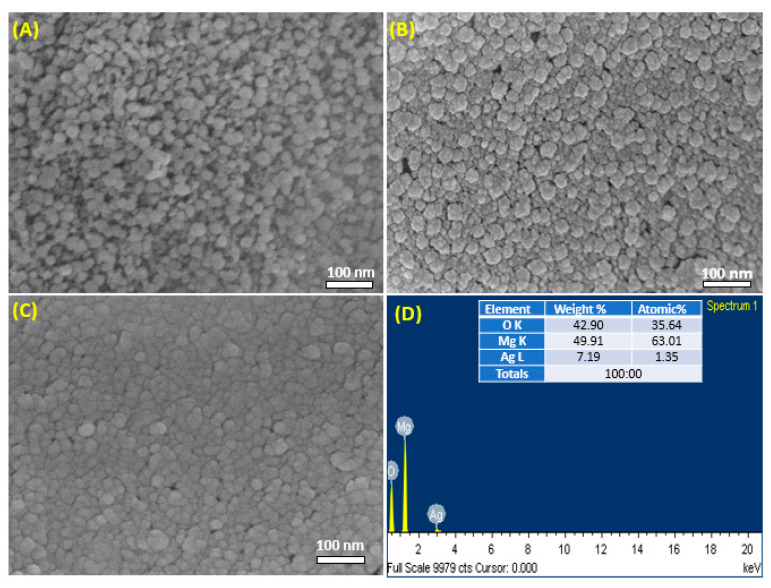
SEM images of pure (**A**), 1% Ag-MgO (**B**), and 7.5% Ag-MgO (**C**). EDX spectra of 7.5% Ag-MgO NPs (**D**).

Surface morphology and elemental composition of prepared samples were further analyzed by scanning electron microscopy (SEM). Figure 4A–C represents the typical SEM images of pure MgO NPs, Ag (1%) MgO NPs, and Ag (7.5%) MgO NPs, respectively. These images indicated spherical morphology with uniform distribution [36]. Figure 4D shows the atomic percentage of Mg, Ag, and O elements of 7.5% Ag-MgO NPs. SEM images suggest a slight difference in the morphology of pure MgO and Ag-MgO NPs [10]. In addition, aggregation polarity and electrostatic attraction of prepared samples leads to agglomeration of NPs [37].

**Figure 5:**
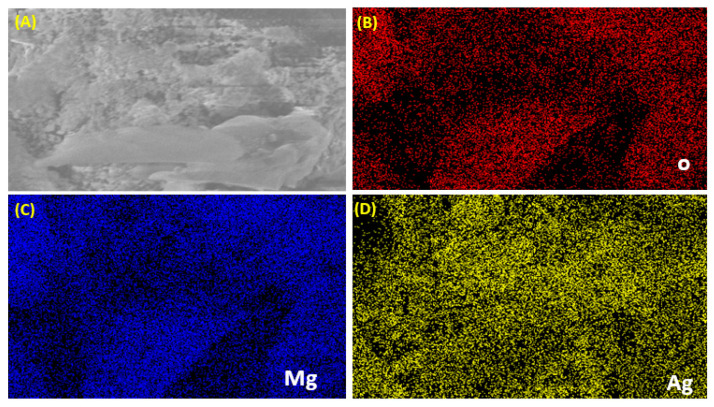
Elemental image mapping of 7.5% Ag-doped MgO NPs (**A**), oxygen (O) (**B**), magnesium (Mg) (**C**), and silver (Ag) (**D**).

Figure 5 shows the elemental mapping of 7.5% Ag-doped MgO NPs. The Mg, Ag, and O elements are uniformly distributed as reported in other studies [38].

### 3.5. X-ray Photoelectron Spectroscopy (XPS) Analysis 

**Figure 6:**
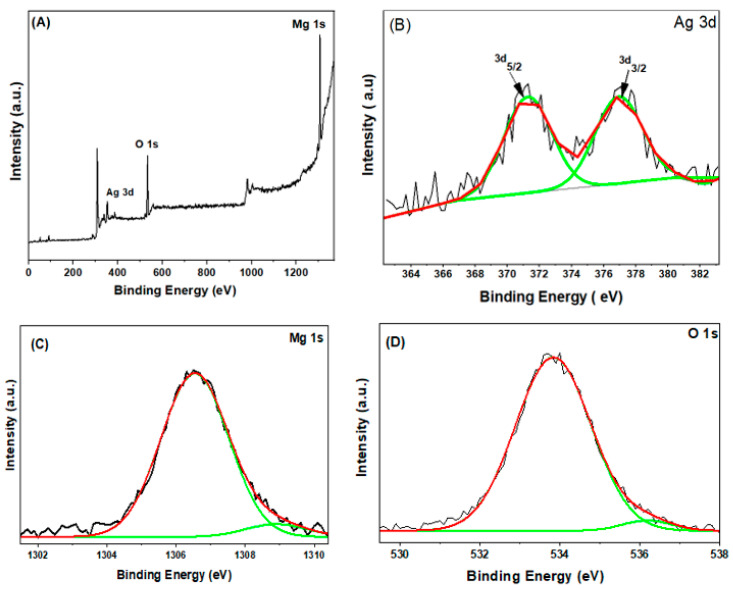
XPS survey spectra for 7.5 % Ag-doped MgO NPs (**A**), Ag 3d (**B**), Mg 1s (**C**), and O1s (**D**).

The chemical composition and elemental status of 7.5% Ag-doped MgO NPs were detected by XPS. Figure 6A shows the signals of magnesium (Mg), silver (Ag), and oxygen (O), which indicate the presence of these elements in the prepared samples. The Ag 3d XPS spectra of 7.5% Ag-doped MgO NPs is illustrated in Figure 6B. The characteristic spin-orbits Ag 3d_5/2_ and Ag 3d_3/2_ are observed at 371.39 eV and 377.09 eV, respectively. This finding proves the existence of Ag and Ag-Mg-O compounds in the synthesized samples [39,40]. Mg 1s XPS spectra (Figure 6C) showed the main and shoulder peaks at 1306.52 eV and 1308.93 eV, respectively, which suggests that Mg ions are present in a +2 oxidation state [38]. Furthermore, Figure 6D illustrates the O1s spectra with two sub-peaks at 533.81 eV and 536.18 eV, respectively. There is a possibility that adsorbed oxygen caused the first peak of O1s, while the second peak of O1s could be attributed to the lattice oxygen of MgO [27,38]. 

### 3.6. Fourier-Transform Infrared (FTIR) Analysis

**Figure 7:**
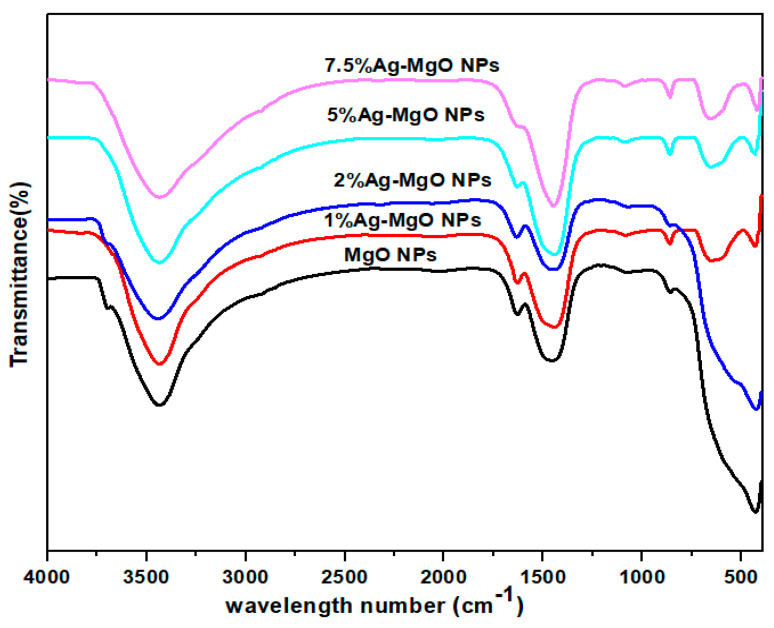
FTIR spectra of pure, 1%Ag -MgO, 2% Ag-MgO, 5% Ag-MgO, and 7.5% Ag-MgO NPs.

The FTIR technique was utilized to investigate the chemical bonds of the prepared samples. Figure 7 shows FTIR spectra of both pure MgO NPs and Ag (1%, 2%, 5%, 7.5 mol%)-MgO NPs. It was recorded at room temperature and covered the wavelength region from 450 cm^−1^ to 4000 cm^−1^. The peak intensity of Ag (1%, 5%, 7.5 mol%)-MgO NPs at 431 cm^−1^ is reduced because of Ag origination on the surface of MgO NPs [41]. The observed peaks in the spectra of pure MgO NPs and Ag-doped MgO NPs are located at 860 cm^−1^, 1450 cm^−1^, 1080 cm^−1^, 1634 cm^−1^, 3420 cm^−1^, and 3704 cm^−1^. The peaks between 431 cm^−1^ to 1080 cm^−1^ can be assigned to Mg-O stretching vibrations [26]. The peaks at 2920 cm^−1^ and 2010 cm^−1^ demonstrate C=O bond because of symmetry and asymmetry vibrations, respectively [42]. For the absorbed water molecules, the peaks of the O-H stretching and bending vibration appeared at 3420 cm^−1^ and 1450 cm^−1^, respectively [43]. However, the peak at 3704 cm^−1^ in Ag-doped MgO NPs disappears with increasing Ag concentration due to creation of Ag-O bonds and the replacement of Mg with Ag [39].

### 3.7. Photoluminescence (PL) Spectroscopy Analysis 

**Figure 8:**
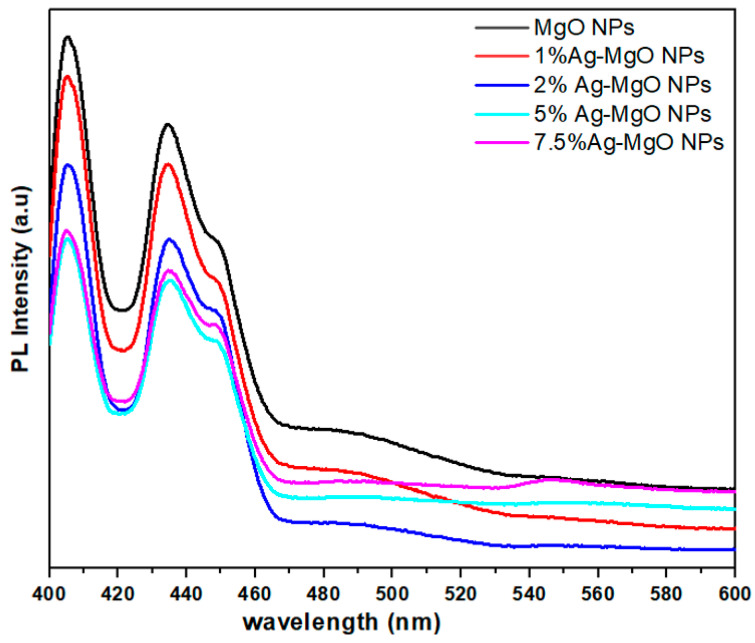
Photoluminescence spectra of pure, 1% Ag-MgO, 2% Ag-MgO, 5% Ag-MgO, and 7.5% Ag-MgO NPs.

The photoluminescence (PL) spectroscope is a significant tool to investigate the electron-hole recombination rate of NPs. Figure 8 illustrates the PL spectra of both pure MgO NPs and Ag (1%, 2%, 5%, 7.5%)-MgO NPs at an excitation wavelength of 385 nm [44]. The peaks at 404 nm, 434 nm, and 450 nm are observed in all samples. However, the intensity of all peaks decreased with increasing concentrations of Ag dopant [45]. Lower intensity of Ag-doped MgO suggests a significant migration of charge carriers (electrons and holes) from the inner part to the surface of NPs; hence, they can then participate in surface redox reactions. This phenomenon is very important for enhanced photocatalytic activity [9].

### 3.8. Photocatalytic Study

**Figure 9:**
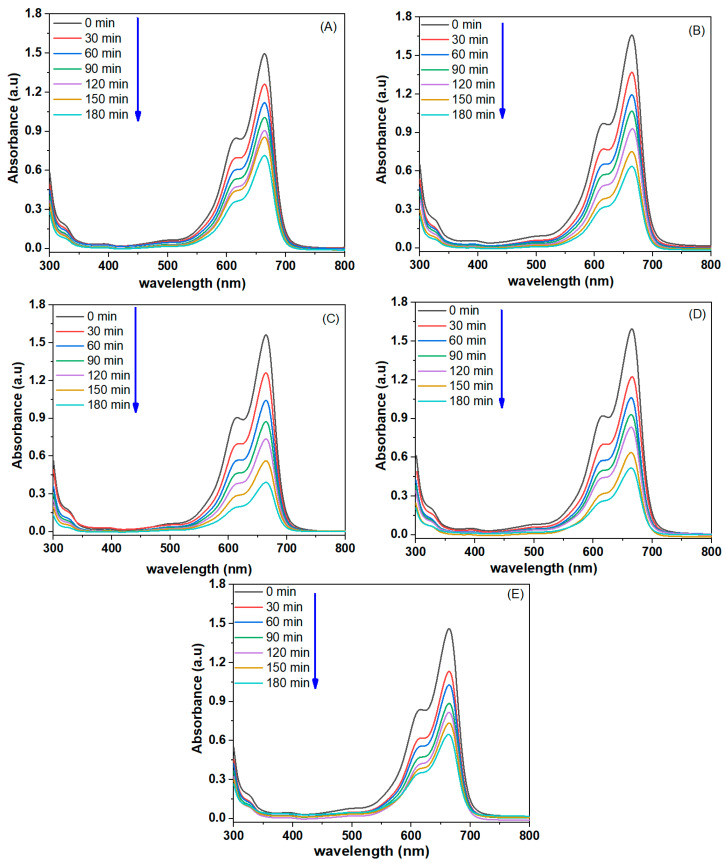
The UV light absorbance of MB solutions as a function of irradiation time (**A**) pure MgO NPs, (**B**) 1% Ag, (**C**) 2% Ag, (**D**) 5% Ag, (**E**) 7.5% Ag-doped MgO NPs.

**Figure 10:**
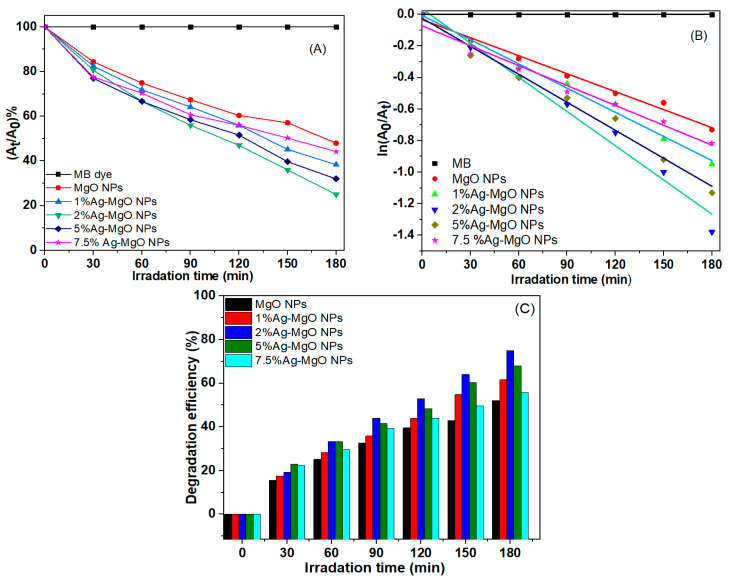
The plot of *A_t_*/*A*_0_ versus time for the degradation of MB using pure MgO NPs and Ag-doped MgO NPs under UV irradiation (**A**), kinetic fit for the degradation of MB (**B**), and the efficiency of the degradation (D%) with a period time in range 30–180 min (**C**).

In the present work, MB dye was used as a model pollutant to measure the photocatalytic activity of pure MgO NPs and Ag (1%, 2%, 5%, 7.5 mol%)-MgO NPs under UV irradiation. Figure 9A–E illustrates the UV–vis absorption spectra of MB dye with pure MgO NPs and Ag-doped MgO NPs. Degradation of MB dye following exposure of synthesized samples was determined by the intensity of the decay of the characteristic absorption peaks around 664 nm as a function in irradiation time (t). Every 30 min, the intensity of the absorption peak was decreased, and the blue MB dye solution turned colorless after 180 min, as shown in this report [46]. As shown in Figure 9A,E, there is a small decrease in the intensity of the absorbance peak after 180 min. A recent study also showed the photocatalytic activity of MgO NPs by degradation efficiency of MB dye under UV irradiation [47]. Figure 10A shows the plot of *A_t_*/*A*_0_ versus time for the degradation of MB dye using pure MgO NPs and Ag-MgO NPs under UV irradiation in the time interval of 30–180 min. Figure 10B also presents the curves relative to ln(*A*_0_/*A_t_*) versus the irradiation time. The photodegradation reactions in the prepared samples are determined by the first-order kinetic rate constant (k). The calculated rate constant (k) of MB dye was 0.0038 min^−1^, 0.0051 min^−1^, 0.0072 min^−1^, 0.0072 min^−1^, 0.0059 min^−1^, and 0.0042 min^−1^ for pure and Ag (1%, 2%, 5%, 7.5%)-MgO NPs, respectively. These values of the rate constant (k) were in agreement with other reports [18,48,49,50]. 

Results suggested that the rate constant of 2% Ag-doped MgO NPs was almost two-fold higher than pure MgO NPs. Figure 10C illustrates the efficiency of MB degradation of pure MgO NPs and Ag-doped MgO NPs. Results showed that the efficiency of MB degradation of the prepared samples was up to 52% to 75% for 180 min, respectively, which is consistent with a previous study [18]. Surprisingly, 2% Ag-doped MgO NPs (Figure 10A) has shown maximum degradation of MB dye as compared to 1%, 5%, and 7.5% Ag-MgO NPs. This could be due to maximum migration of charge carriers for 2% Ag doping at the surface of MgO NPs. Higher photocatalytic activity after 2% Ag doping in MgO NPs was also reported in other studies [20,22,51,52,53]. Table 2 shows the comparison of degradation efficiency for various photocatalysts on MB dye. These results show that Ag dopant ions can impact the charge separation and charge carrier-recombination/migration [54]. 

**Figure 11:**
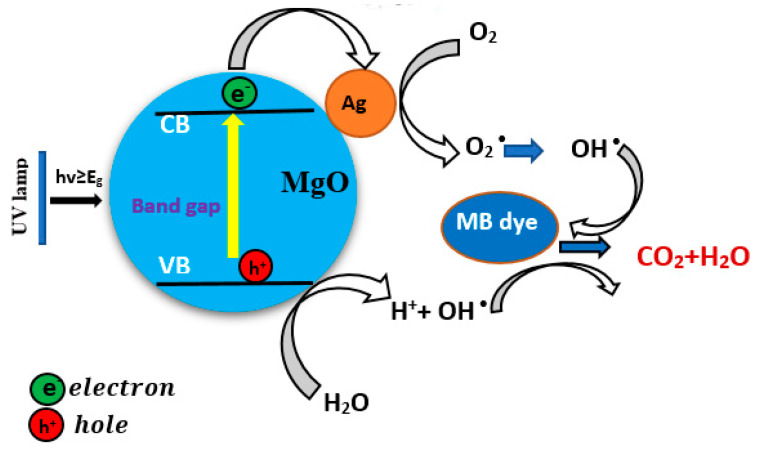
Diagram mechanism of photocatalytic process of Ag-doped MgO NPs.

The possible mechanisms of photocatalytic activity of Ag-doped MgO NPs is proposed in Figure 11. Upon UV irradiation, electrons in the valance band (VB) of pure MgO NPs are excited to the conduction band (CB), leaving the same number of holes in the valance band (Equation (3)). The excited electrons can be easily trapped by the Ag dopant and release superoxide radicals ($O_{2\;}{}^{●}$) (Equation (4)). Similarly, generated holes in the valance band (VB) cannot attack compounds on surface of materials as well as oxidize water molecules to form hydroxyl radicals (${OH}^{●}$) (Equation (5)). Following that, the organic contaminants (MB dye) might be degraded by reactive agents ($O_{2\;}{}^{●},{OH}^{●}$) or be immediately destroyed by holes (*h^+^*) (Equation (6)).

(3) $$MgO+hv\to \;e^{-}+h^{+\;}$$

(4) $$e^{-\;}+O_{2\;}\to \;O_{2\;}{}^{●}$$

(5) $$h^{+}+H_{2}O\to OH{}^{●}+H^{+}$$

(6) $$\left(h^{+},\;O_{2}{}^{●},OH{}^{●}\right)+\mathrm{organic}\;\left(\mathrm{MB}\;\mathrm{dye}\right)\to CO_{2}+H_{2}O$$

This mechanism reduced electron-hole recombination, resulting in an improvement in the photocatalytic efficiency of the prepared samples. The improved photocatalytic activity of photogenerated electron-hole pairs in the prepared samples was also presented by the PL spectra (Figure 8). Consequently, photogenerated charge carriers can be separated effectively and were observed to have greater photocatalytic activity of Ag (2%)-doped MgO NPs than those of pure MgO NPs using MB dye [13,18].

### 3.9. Cytotoxicity Assay

**Figure 12:**
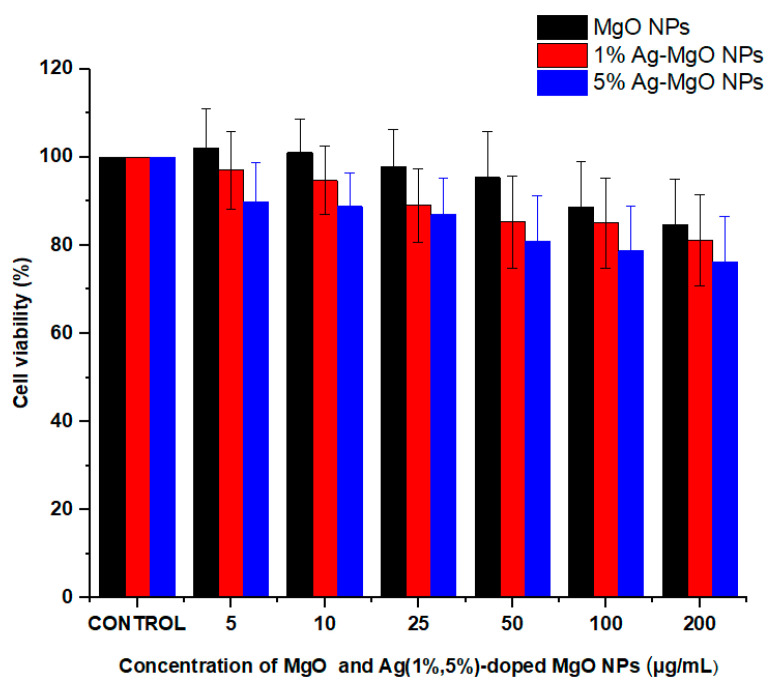
Cytotoxicity of pure and Ag (1%, 5%)-doped MgO NPs in HUVECs by MTT assay.

It is important to examine the cytotoxicity of photo-catalyst materials before their application in environmental remediation [34,61]. In this study, the MTT assay was applied to examine the cytotoxicity of pure and Ag-doped MgO NPs on human normal umbilical vein endothelial cells (HUVECs) (Figure 12). Cells were exposed to different concentrations (0, 5, 10, 25, 50, 100, and 200 μg/mL) of pure MgO NPs and Ag (1%, 5%) MgO NPs for 24 h. Results showed that pure and Ag-doped MgO NPs did not induce significant cytotoxicity to HUVE cells. These results suggested that Ag-doped Mg NPs have the potential to be applied for photocatalysis without generating toxicity to human cells [36,62].

## 4. Conclusions

A simple sol-gel method was applied to synthesize pure and Ag (1%, 2%, 5%, 7.5 mol%)-doped MgO NPs by using magnesium nitrate hexahydrate and silver nitrate as precursors. XRD spectra confirmed the formation of Ag-doped MgO NPs. Ag peaks appear in Ag-doped MgO NPs because of the higher ionic radii of Ag from Mg. However, the crystallite size of MgO NPs decreased from 12 nm to 9 nm due to increasing concentration of Ag. TEM illustrated that agglomeration of the particles increases with an increasing of Ag due to the strong interactions between nanoparticles. EDX results indicated the presence of Ag, Mg, and O elements in Ag-doped MgO NPs. FTIR indicated the presence of O-H stretching and formation of Ag-O bonds. PL results showed that the intensity of the excitation and emission peaks decreased with an increasing concentration of Ag because of Ag ions on the surface of the MgO NPs. Ag-MgO NPs exhibited outstanding photocatalytic activity compared to the pure MgO for the degradation of the MB under UV light. The results show that 2% Ag-doped MgO NPs can photodegrade MB dye 75% in 180 min of irradiation time under UV illumination. MTT assay showed that pure and Ag-doped MgO NPs were not toxic to human normal HUVE cells. This warrants further study on photocatalysis and biomedical applications of Ag-doped MgO NPs.

## Figures and Tables

**Table 1:** Structure characterization of pure and Ag-MgO NPs.

Sample	XRD Size(nm)	TEM Size (nm)
MgO	12 nm	14.3 nm
1% Ag-MgO	11.5 nm	13.3 nm
2% Ag-MgO	11 nm	12 nm
5% Ag-MgO	9.5 nm	10.5 nm
7.5% Ag-MgO	9 nm	9.5 nm

**Table 2:** Comparison of degradation efficiency for various samples on MB dye.

Samples	Degradation Efficiency (%)	Source of Light	References
Ag/Ag_2_O NPs	84.50%	UV–vis light irradiation	[55]
Fe/MgO NPs	88%	UV–vis light irradiation	[18]
Ag/ZnO NPs	96%	UV–vis light irradiation	[56]
ZnO/MgO nanocomposite	91%	UV–vis light irradiation	[57]
Ag/TiO_2_/CNT NPs	50%	Artificial light	[58]
Zn/In_2_O_3_ NPs	81%	UV–vis light irradiation	[59]
MgO/ZnO/In_2_O_3_ nanocomposite	80%	UV–vis light irradiation	[60]
Ag-MgO NPs	75%	UV–vis light irradiation	Present work
